# Multiple paragangliomas: a case report

**DOI:** 10.1186/s12920-020-00789-8

**Published:** 2020-09-18

**Authors:** Vladislav S. Pavlov, Dmitry V. Kalinin, Elena N. Lukyanova, Alexander L. Golovyuk, Maria S. Fedorova, Elena A. Pudova, Maria V. Savvateeva, Anastasiya V. Lipatova, Zulfiya G. Guvatova, Andrey D. Kaprin, Marina V. Kiseleva, Tatiana B. Demidova, Sergey A. Simanovsky, Nataliya V. Melnikova, Alexey A. Dmitriev, George S. Krasnov, Anastasiya V. Snezhkina, Anna V. Kudryavtseva

**Affiliations:** 1 Engelhardt Institute of Molecular Biology, Russian Academy of Sciences, 32 Vavilova str, Moscow, 119991 Russia; 2 Vishnevsky Institute of Surgery, Ministry of Health of the Russian Federation, 27 Bol’shaya Serpukhovskaya str, Moscow, 117997 Russia; 3 National Medical Research Radiological Center, Ministry of Health of the Russian Federation, 3 2nd Botkinski drive, Moscow, 125284 Russia; 4 A. N. Severtsov Institute of Ecology and Evolution, Russian Academy of Sciences, 33 Leninskij prosp, Moscow, 119071 Russia

**Keywords:** Multiple paragangliomas, Carotid and vagal paragangliomas, *SDHx*, Germline and somatic mutations, High-throughput exome sequencing, Immunohistochemistry, Case report

## Abstract

**Background:**

Carotid and vagal paragangliomas (CPGLs and VPGLs) are rare neoplasms that arise from the paraganglia located at the bifurcation of carotid arteries and vagal trunk, respectively. Both tumors can occur jointly as multiple paragangliomas accounting for approximately 10 to 20% of all head and neck paragangliomas. However, molecular and genetic mechanisms underlying the pathogenesis of multiple paragangliomas remain elusive.

**Case presentation:**

We report a case of multiple paragangliomas in a patient, manifesting as bilateral CPGL and unilateral VPGL. Tumors were revealed via computed tomography and ultrasound study and were resected in two subsequent surgeries. Both CPGLs and VPGL were subjected to immunostaining for succinate dehydrogenase (SDH) subunits and exome analysis. A likely pathogenic germline variant in the *SDHD* gene was indicated, while likely pathogenic somatic variants differed among the tumors.

**Conclusions:**

The identified germline variant in the *SDHD* gene seems to be a driver in the development of multiple paragangliomas. However, different spectra of somatic variants identified in each tumor indicate individual molecular mechanisms underlying their pathogenesis.

## Background

Paragangliomas of the head and neck (HNPGLs) are rare neuroendocrine tumors [1]. There are several common paraganglioma localizations corresponding to the locations of paraganglia from which they are formed. Carotid paragangliomas (CPGLs) are most common, followed by middle ear paragangliomas, vagal (VPGL), and laryngeal paragangliomas [2]. These tumors are highly hereditary and associated with the germline mutations in known susceptibility genes, including *SDHx*, *SDHAF2*, *TMEM127*, *MAX*, and others [3]. Mutations in these genes predispose to different forms of paragangliomas (early, syndromic, multiple, and malignant).

HNPGLs commonly develop as single unilateral tumors, with only 1% of sporadic cases being multiple [4]. However, the number of multiple HNPGLs greatly increases in familial tumors. As multiple paragangliomas are rare, every case is important to study for a better understanding of genetics and molecular mechanisms causing their initiation and progression.

## Case presentation

The case below describes multiple paragangliomas diagnosed in a Russian woman, presenting as two CPGLs at both sides of the neck and one VPGL. The aim of the study was to investigate the molecular mechanisms underlying the development of multiple paragangliomas by examining clinical and pathological characteristics along with the genetic variations of the three tumors.

**Fig. 1:**
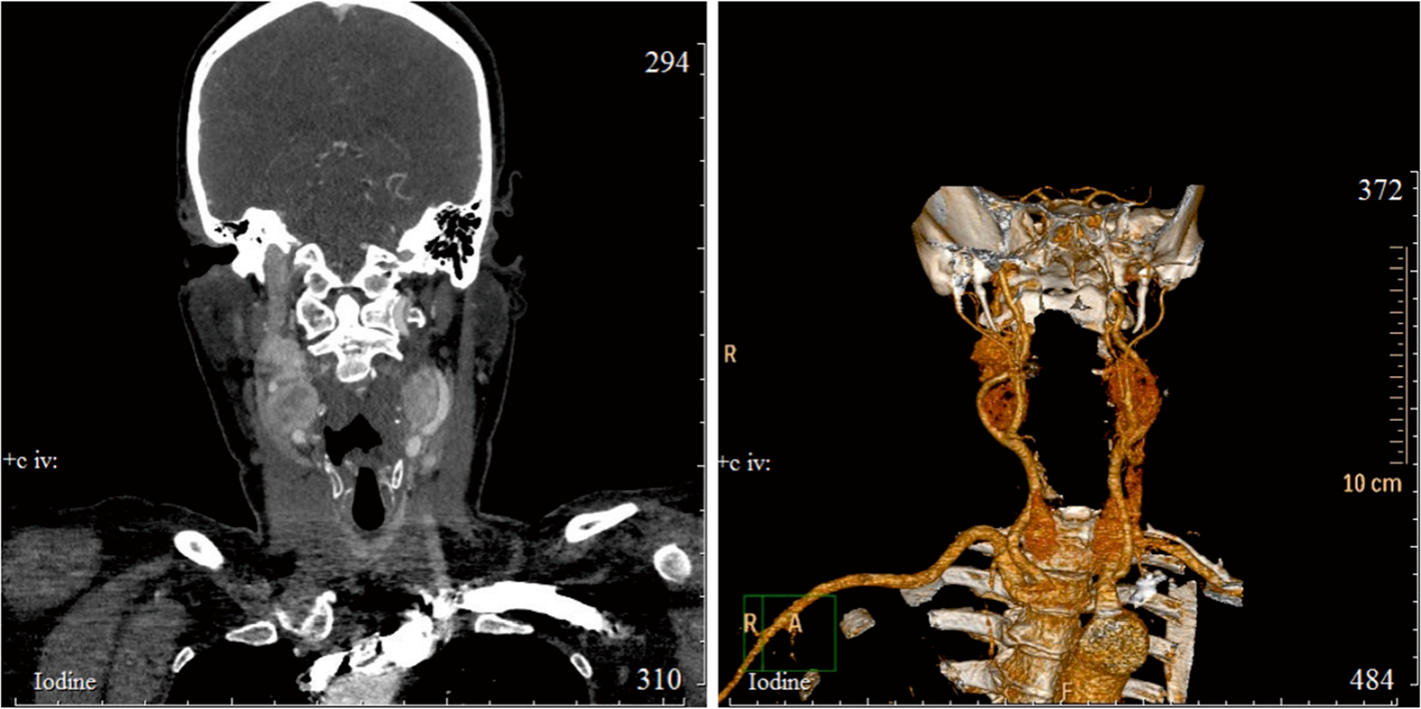
Computed tomography of the patient's head and neck before surgery. CT scan (left); 3D reconstruction (right)

A 50-year-old female was diagnosed with extravascular compression of the carotid arteries and CPGLs on both sides of the neck. Clinical symptoms include arterial hypertension and painless rounded masses. Computed tomography (CT) and ultrasound (US) study revealed the presence of tumors in the areas of the carotid bifurcation, solid neoplasia 32 × 25 mm on the left side of the neck and two-nodal tumor 46 × 24 mm on the right side of the neck, respectively. These neck masses were heterogeneous in structure and predominantly hypoechoic and hypervascular*.* The CT study with contrast also revealed the presence of hypointense, highly vascularized masses at the right and left carotid bifurcations (Fig. 1).

**Fig. 2:**
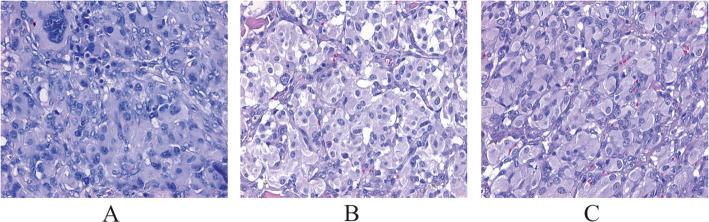
Hematoxylin-eosin (H&E) staining of carotid and vagal paragangliomas. **a** left CPGL, **b** right CPGL, and **c** right VPGL. Specific “Zellballen” growth pattern of paragangliomas (small nests of chief cells with pale eosinophilic staining, surrounded by supporting sustentacular cells) can be seen

The patient was subjected to surgery for the left tumor resection. At the time of surgery, the lower pole of the hypervascularized tumor was located below the outer carotid artery (OCA) bifurcation, spreading along the carotid arteries in the proximal direction and wrapping around the posterior, anterior, and lateral surfaces. Bifurcation of carotid arteries was involved in the tumor mass. The upper pole of the tumor was associated with the vagus nerve. The tumor (25 × 2 × 17 mm) was completely removed and subjected to pathological evaluation. The patient was discharged with a planned re-hospitalization to remove the tumor on the right. Histological examination of the resected tumor confirmed carotid paraganglioma (Fig. 2). Hematoxylin-eosin (H&E) staining showed a Zellballen structure that is typical for paragangliomas. Chief tumor cells exhibited positive staining for chromogranin A, synaptophysin, and CD56 antibodies indicating a neuroendocrine tumor. S100 protein was expressed in sustentacular cells. Tumor cells were negative for cytokeratin AE1/AE3.

A surgery on the right side of the neck was performed after a year. As per the US study reports, VPGL and enlarged lymph node were primarily detected. During the surgery, lymph node (15 × 5 mm) above the carotid artery bifurcation was removed and further subjected to histological examination for metastases. In addition, tumor-like mass (35 × 20 mm), laterally suppressing the internal carotid artery (ICA) was observed directly at carotid artery bifurcation and resected. After the tumor resection, one more tumor-like formation (60 × 20 mm) originating from the vagus nerve was visualized under it and removed. The vagus nerve was resected and ligated. Both the tumors were histologically examined and displayed paragangliomas with no lymph node metastases (Fig. 2).

For all the tumor samples from the patient (left and right CPGLs, VPGL), immunohistochemistry (IHC) analysis of succinate dehydrogenase (SDH) subunit expression was performed (Additional file 1). SDH complex consists of four subunits (SDHA, SDHB, SDHC, and SDHD) encoded by the corresponding genes [5, 6]. Germline and somatic mutations in the *SDHx* genes are commonly associated with paragangliomas/pheochromocytomas [7, 8]. Immunohistochemistry for SDH subunits is a valuable additional tool in the histopathological study of paragangliomas that is used in the clinic for assessment of SDH loss, which can be associated with the pathogenic mutations in any *SDHx* genes.

Immunoreactions for SDH subunits were carried out using primary antibodies from Abcam (USA) for each SDH subunit: SDHA, monoclonal, clone 2E3GC12FB2AE2; SDHB, monoclonal, clone 21A11AE7; SDHC, monoclonal, clone EPR11035(B); SDHD, polyclonal. We found weak diffuse weak SDHB staining in VPGL and both the left and right side CPGLs. According to the literature, weak diffuse staining of the SDHB subunit can reflect pathogenic mutations in any SDHx genes. We detected weak diffuse SDHB staining in all studied tumors indicating the presence of germline pathogenic mutation in one of the *SDHx* genes in the patient.

Additionally, we carried out the exome-sequencing of three tumors, lymph node, and blood from the patient. The DNA from tumors and lymph node was extracted with a High Pure FFPET DNA Isolation Kit (Roche, Switzerland). The DNA was isolated from blood cells using a MagNA Pure Compact Nucleic Acid Isolation Kit I (Roche) on a MagNA Pure Compact Instrument (Roche). Exome libraries were prepared with the Rapid Capture Exome Kit (left CPGL) and TruSeq Exome Library Prep Kit (right CPGL and VPGL) from Illumina (USA). High-throughput exome sequencing was performed on a NextSeq 500 System (Illumina) under a paired-end mode of 76 × 2 bp for tumors and lymph node, and 156 × 2 bp for blood with 300x minimum coverage. The exome sequencing data of paragangliomas are available in the NCBI SRA under the accession numbers PRJNA411769 (left CPGL, Pat01), PRJNA476932 (right CPGL, Pat104), and PRJNA561073 (VPGL, Pat6). Bioinformatic analysis is described in our previous study [9]. Missense variants were considered as likely pathogenic if they were predicted by at least three prediction tools and characterized by conservation score ≥ 0.5.

Exome analysis revealed a likely pathogenic germline missense variant in the *SDHD* gene, NM_003002.3: c.305A > G, p.H102R (chr11: 111959726, rs104894302). Pathogenic/likely pathogenic germline variants in other genes, for which the association with paragangliomas/pheochromocytomas has been shown, were not found.

Identified likely pathogenic somatic variants were different for each tumor (Additional file 2). In left CPGL, we found missense likely pathogenic somatic variants in two genes, *TENM3* [NM_001080477: c.C5082A, p.N1694K (chr4: 183696084)] and *EPHA5* [NM_004439: c.G682A, p.V228I (chr4: 66467587)].

In right CPGL, a variety of likely pathogenic variants (stop-gain, frameshift, and missense) were detected. Stop-gain variants were found in *NRXN3* [NM_004796: c.C1387T, p.Q463X (chr14: 79432478)] and *RELN* [NM_005045: c.C9052T, p.R3018X (chr7: 103137114)], missense variants were revealed in *TRIP12*, *JAG1*, *ASXL1*, *LMBRD*1, *DHX9*, *AASS*, and *TP53*. For the *TP53* gene, we found two mutations: a pathogenic/likely pathogenic variant, NM_001126115: c.A446T, p.D149V (chr17: 7577096, rs587781525), that has been reported in ClinVar, and a previously undescribed likely pathogenic variant, NM_000546: c.A170G, p.D57G (chr17: 7579517).

In the case of VPGL, we found a pathogenic variant in mtDNA (MT: 3243, rs199474657) and likely pathogenic missense, frameshift and stop-gain variants in a number of genes (*LRP1, SPEN, PPP4R1*, *XPO6*, *FBN1*, *C1QB*, and others) (Additional file 2).

## Discussion

We found a germline pathogenic variant in the *SDHD* gene in the patient. According to the literature, *SDHD* mutations are frequently associated with multiple paragangliomas. A study of 176 patients with HNPGLs divulged multiple paragangliomas in 33 individuals (18.9%) [10]. *SDHx* mutations were found in 34 patients, of which 22 were diagnosed with multiple paragangliomas. Moreover, 18 out of 22 patients with multiple paragangliomas had mutations in *SDHD*, 1 patient exhibited *SDHB* mutation, and 3 patients carried variations in the *SDHC* gene.

In a recent report, multiple synchronous or metachronous HNPGLs were found in 79 out of 147 patients studied (54%) [11]. A group of patients (98/147) were tested for the status of *SDHx* mutations; 74 patients carried mutations in either *SDHB* (10/74) or *SDHD* (64/74) genes. *SDHB* mutations were found in two cases of multiple paragangliomas, whereas 56 had mutated *SDHD.* Notably, p.Asp92Tyr mutation in the *SDHD* gene (one of the Dutch founder mutations) was the most prevalent variant identified in 50% of *SDHD* mutation carriers (32/64).

Another study on *SDHB*, *SDHC*, and *SDHD* gene mutation analysis in a large cohort of patients with a personal or family history of paragangliomas/pheochromocytomas was performed [12]. From the 1832 individuals tested, 876 patients carried mutations in either *SDHB* (673), *SDHC* (43), or *SDHD* (160). In summary, results from all the studies indicate a high frequency of *SDHx* mutations in paragangliomas. Moreover, most multiple paragangliomas are associated with *SDHx* variants, predominantly *SDHD* mutations, which are in accordance with our results.

The variant in the *SDHD* gene identified in the study has been described only once in a malignant paraganglioma [13]. Thus, the mutation seems to be quite rare; although, at the same position, histidine replaced with leucine, proline, or tyrosine was found in several cases of hereditary paragangliomas [14–16].

We observed different spectra of somatic mutations in three tumors studied. In left CPGL, only two genes, *TENM3* and *EPHA5* with likely pathogenic somatic variants were identified. Mutations in these genes have first been found in paragangliomas/pheochromocytomas. *TENM3* and *EPHA5* encode for proteins with important functions in neuronal cells and have been shown to be involved in tumorigenesis [17–20].

In right CPGL, a greater number of likely pathogenic somatic variants were revealed; however, no variants were found in the genes mutated in left CPGL. Stop-gain variants were determined in *NRXN3* and *RELN* encoding for proteins that are involved in cell adhesion and cell-cell interactions in neural cells, respectively. Both genes were shown to be associated with glioblastoma pathogenesis [21–23] and variants in these genes were also detected in a number of tumors [24–26]. We also found two variants in the *TP53* gene in the right CPGL. One variant was previously reported in ClinVar as a pathogenic mutation, the other variant was first detected in ourstudy. The presence of these two variants in *TP53* can enhance the deleterious effect on protein function. More missense likely pathogenic mutations and frameshift variants were identified in the genes encoding for proteins that participate in important cellular processes, such as ubiquitin fusion degradation and regulation of DNA repair (*TRIP12*, a tumor suppressor gene), mediation of Notch signaling (*JAG1*), and transcriptional regulation (*DHX9*, *KLF12*, and *KAT6B*). Several genes with frameshift mutations are highly expressed in neural cells and involved in the neurite growth (*PLPPR5*) and the brain development (*KAT6B* and *ADGRV1*).

In VPGL, we identified variants that have not been found in the left and right CPGLs in the patient. Stop-gain mutations were revealed in the genes involved in the regulation of cell cycle (*LIN54*) and transcription (*ZNF717*), and in the *CCDC82* gene, encoding for protein with unknown function. Variants in *ZNF717* were previously observed in gastric, colorectal, and hepatocellular cancer [27–29]. Missense mutations in the genes shown to be involved in tumorigenesis that were predicted as likely pathogenic by a majority of prediction tools (*C1QB*, *XPO6*, *PPP4R1*, *PPIP5K2*, and *LRP1),* were also found in VPGL [30–34]. Frameshift mutations which usually result in nonfunctional protein were observed in many genes. In addition, we detected mutation in mtDNA that was reported in ClinVar as a germline or somatic pathogenic variant associated with mitochondrial diseases.

## Conclusion

We identified a likely pathogenic germline variant in the *SDHD* gene and likely pathogenic somatic variants in a number of genes in the patient with multiple paragangliomas (left and right CPGLs, and VPGL). Somatic variants differed amongst the tumors. Thus, we assume that the variant found in the *SDHD* gene is a driver mutation and its co-occurrence with other mutations in each of the three tumors can lead to the development of paraganglioma via different molecular pathways.

## Supplementary information

**Additional file 1.** Immunohistochemical staining of SDH subunits in carotid and vagal paragangliomas.

**Additional file 2.** Pathogenic/likely pathogenic somatic variants in carotid and vagal paragangliomas.

## Data Availability

All data generated or analyzed in this study are included in the published article. The exome sequencing data of carotid paragangliomas are available in the NCBI SRA under the accession numbers PRJNA411769 and PRJNA476932. The sequencing data of vagal paraganglioma are available in the NCBI SRA under the accession number PRJNA561073.

## Abbreviations

HNPGL: Head and neck paraganglioma
CPGL: Carotid paraganglioma
VPGL: Vagal paraganglioma
CT: Computed tomography
US: Ultrasound study
CCA: Common carotid artery
ICA: Internal carotid artery
OCA: Outer carotid artery
