# Supplementary material for: Multiple paragangliomas: a case report
Source: BMC Med Genomics. 2020 Sep 18;13(Suppl 8):125. doi: 10.1186/s12920-020-00789-8 (PMC7500000; doi:10.1186/s12920-020-00789-8)

Immunohistochemical staining of SDH subunits in carotid and vagal paragangliomas

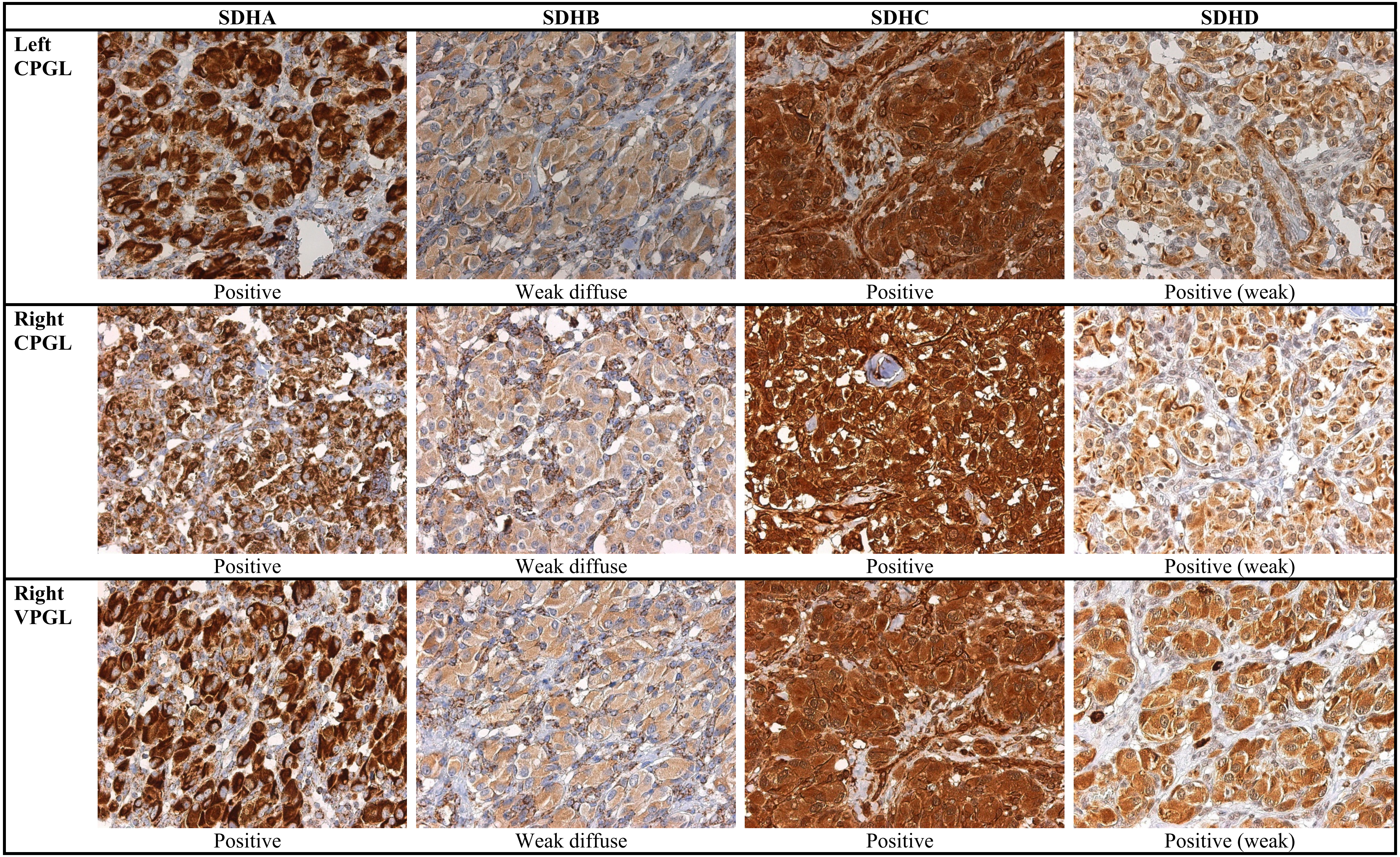

Supplement: Supplementary file 1 — Additional file 1. Immunohistochemical staining of SDH subunits in carotid and vagal paragangliomas. [file 12920_2020_789_MOESM1_ESM.pdf]
